# 

**Published:** 1975-01

**Authors:** 


The
Bristol Medico-Chirurgical Journal
(Incorporating the Medical Journal of the South-West)
Established 1883

Vol. 90 (i) JANUARY 1975 No. 334

### BRISTOL MEDICO-CHIRURGICAL JOURNAL

**Editor: Dr. D. S. Reeves**

**Published Quarterly** **Annual Subscription £2 Post Free**

### BRISTOL MEDICO-CHIRURGICAL SOCIETY

**President 1974/75:** Dr. J. Apley, C.B.E.

**President-elect:** D. C. Bodenham, Esq.

**Honorary Secretary:** A. J. Webb, Esq., 7 Percival Road, Bristol 8.

**Honorary Treasurer:** Dr. Frank Ross, Bristol Royal Infirmary, Bristol 2.

The Society was founded in 1874. In 1883 publication of the Journal began and has continued quarterly ever since then. During the years 1949 to 1962 it appeared under the title of "The Medical Journal of the South West".

### THE BRISTOL MEDICO-CHIRURGICAL JOURNAL

**Editorial Committee**

Editor Dr. D. S. Reeves, Southmead Hospital, Bristol.

Members Dr. Rhys Davies.
Dr. M. B. Lennard.
Professor R. C. Wofinden.
Dr. D. W. Wright.
The Hon. Treasurer and Hon. Secretary of the Society.

Secretary Mr. B. P. Jones, The Library, Medical School, University Walk, Bristol BS8 1TD.

### NOTICE TO CONTRIBUTORS

The Journal is especially concerned to provide a place for the recording of medical thought, interests, and practice in and around Bristol. It therefore welcomes articles that, as well as being of immediate interest to members, will document the local and contemporary medical scene for our successors.

Original articles are invited provided that they have not been published elsewhere. References in the text should be by author's name, with year of publication in parenthesis; they should be listed at the end in alphabetical order, the name of each journal or book being given in full—**not abbreviated**—and the title of the article added. Illustrations should be supplied ready for reproduction. Line drawing should be in Indian ink on firm white paper or board. The author will be informed if it is found necessary to ask him to pay for the making of blocks.

The following contributions will be especially welcome; notes of forthcoming events, meetings held, degrees conferred, staff appointments, honours and public appointments and other local news.

